# General Movements in preterm infants undergoing craniosacral therapy: a randomised controlled pilot-trial

**DOI:** 10.1186/s12906-016-0984-5

**Published:** 2016-01-13

**Authors:** Wolfgang Raith, Peter B. Marschik, Constanze Sommer, Ute Maurer-Fellbaum, Claudia Amhofer, Alexander Avian, Elisabeth Löwenstein, Susanne Soral, Wilhelm Müller, Christa Einspieler, Berndt Urlesberger

**Affiliations:** 1 Division of Neonatology, Department of Paediatrics and Adolescent Medicine, Medical University of Graz, Auenbruggerplatz 34/2, Graz, 8036 Austria; 2 Institute of Physiology, Center for Physiological Medicine & iDN- interdisciplinary Developmental Neuroscience, Medical University of Graz, Graz, Austria; 3 Center of Neurodevelopmental Disorders, Department of Women’s and Children’s Health, Karolinska Institutet, Stockholm, Sweden; 4 Institutes for Medical Informatics, Statistics and Documentation, Medical University of Graz, Graz, Austria; 5 Physiotherapy Unit, University Children Hospital Graz, Graz, Austria

**Keywords:** Infant Development, Neurologic Examination, General movements, Complementary therapies, Preterm Infants, Neonatal intensive care unit, craniosacral therapy, osteopathic manipulative treatment (OMT)

## Abstract

**Background:**

The objective of this study was to investigate neurological short-term effects of craniosacral therapy as an ideal form of osteopathic manipulative treatment (OMT) due to the soft kinaesthetic stimulation.

**Methods:**

Included were 30 preterm infants, with a gestational age between 25 and 33 weeks, who were admitted to the neonatal intensive care unit of the University Hospital of Graz, Austria. The infants were randomized either into the intervention group (IG) which received standardised craniosacral therapy, or the control group (CG) which received standard care. To guarantee that only preterm infants with subsequent normal neurodevelopment were included, follow up was done regularly at the corrected age (= actual age in weeks minus weeks premature) of 12 and 24 months. After 2 years 5 infants had to be excluded (IG; *n* = 12; CG: *n* = 13).

General Movements (GMs) are part of the spontaneous movement repertoire and are present from early fetal life onwards until the end of the first half year of life. To evaluate the immediate result of such an intervention, we selected the General Movement Assessment (GMA) as an appropriate tool. Besides the global GMA (primary outcome) we used as detailed GMA, the General Movement Optimality Score (GMOS- secondary outcome), based on Prechtl’s optimality concept. To analyse GMOS (secondary outcome) a linear mixed model with fixed effects for session, time point (time point refers to the comparisons of the measurements before vs. after each session) and intervention (IG vs. CG), random effect for individual children and a first order autoregressive covariance structure was used for calculation of significant differences between groups and interactions. Following interaction terms were included in the model: session*time point, session*intervention, time point*intervention and session*time point*intervention. Exploratory post hoc analyses (interaction: session*time point*intervention) were performed to determine group differences for all twelve measurement (before and after all 6 sessions) separately.

**Results:**

Between groups no difference in the global GMA (primary outcome) could be observed.

The GMOS (secondary outcome) did not change from session to session (main effect session: *p* = 0.262) in the IG or the CG. Furthermore no differences between IG and CG (main effect group: *p* = 0.361) and no interaction of time*session could be observed (*p* = 0.658). Post hoc analysis showed a trend toward higher values before (*p* = 0.085) and after (*p* = 0.075) the first session in CG compared to IG. At all other time points GMOS were not significantly different between groups.

**Conclusion:**

We were able to indicate that a group of “healthy” preterm infants undergoing an intervention with craniosacral therapy (IG) showed no significant changes in GMs compared to preterm infants without intervention (CG). In view of the fact that the global GMA (primary outcome) showed no difference between groups and the GMOS (detailed GMA-secondary outcome) did not deteriorate in the IG, craniosacral therapy seems to be safe in preterm infants.

**Trial registration:**

German Clinical Trials Register DRKS00004258.

## Background

Both the management and outcome of preterm newborns have changed in the post surfactant era. Due to improvements in survival rates, the focus of neonatal care has shifted to optimizing growth, neurodevelopment and long-term outcomes [1]. However, the stressful environment of a neonatal intensive care unit may compromise these vulnerable infants, in addition to the physiologic consequences of preterm birth.

There is increasing evidence that massage therapy and/or tactile/kinaesthetic stimulation (e.g. osteopathic manipulative treatment (OMT)) improves i) weight gain [2], ii) motor- and neurodevelopmental outcome [3], iii) reduces length of hospital stay (LOS) [4], iv) increases bonding and attachment behaviour in preterm infants [5]. OMT is defined as “the therapeutic application of manually guided forces by an osteopathic physician to improve physiologic function and/or support homeostasis that has been altered by somatic dysfunction” [6, 7]. The OMT techniques used in preterm infants include i) myofascial release, ii) balanced ligamentous/membranous tension, iii) indirect fluidic, and iv) v-spread [8, 9]. Overall, OMT refers to manipulative techniques ranging from articulatory to visceral manipulation including cranial osteopathy [10, 11].

Craniosacral therapy was developed out of OMT by John Upledger, based on the research by William Garner Sutherland [12], who hypothesized that dural tension and decrease of cerebrospinal fluid flow could correlate with a reduction in palpability of the cranial rhythmic impulse [13]. Further it is assumed, that these conditions may be corrected by gentle manipulation of the cranium and sacrum [14].

To evaluate the immediate result of an intervention on the preterm brain, appropriate neuromotor assessment is essential.

General Movements (GMs) are the most frequent, complex and longest lasting pattern of the prenatal and neonatal motor repertoire. They can be observed from a postmenstrual age of 9 weeks to a post term age of 5 months [15]. A systematic review of neuromotor assessment of preterm infants showed that the Test of Infant Motor Performance and the General Movement Assessment (GMA) are the only tools to appropriately assess neuromotor development at term equivalent [16]. The GMA, a tool to delineate the integrity of the young nervous system, focusses on endogenously generated – i.e. without sensory input – age-specific motor patterns [17, 18]. The predictive power of the GMA is equivalent to MRI (white matter assessment) and superior to cranial ultrasound or neurological examination [19, 20].

As very low birth weight infants are extremely small and sensitive to touch, we considered (a) craniosacral therapy as an ideal form of OMT by soft kinaesthetic stimulation and (b) the non-intrusive GMA as the appropriate tool to evaluate its implications. The aim of our study was to investigate neurological short-term effects of craniosacral therapy as a ideal form of OMT for preterm infants.

## Methods

### Participants

Preterm infants, with a gestational age (GA) [21] between 25 and 33 weeks who were admitted to the neonatal intensive care unit of the University Hospital of Graz, Austria, were eligible. As we planned a treatment period of 3 weeks, and most infants are discharged with a GA of 37 weeks, the upper limit of inclusion was 33 weeks. Infants with congenital anomalies, presence of major malformations, any abnormality in cranial ultrasound, elevated bilirubin levels, and any need of respiratory support (need for oxygen or mechanical ventilation) during the study period were excluded. To guarantee that only preterm infants with subsequent normal neurodevelopment were included, follow up was done regularly at the corrected age [21] of 12 and 24 months. The neurological examination was performed according to Touwen [22], for neurodevelopmental testing the Bayley Scales of Infant Development were applied [23]. Some infants that were initially included were excluded later on due to signs of abnormal neurological development. Thus the inclusion of only neurological healthy infants was guaranteed. The ethics committee of the Medical University of Graz approved the study and written parental consent was obtained prior inclusion (20-009ex08/09). The trial was registered with the German Clinical Trials Register DRKS00004258.

### Protocol

**Fig. 1:**
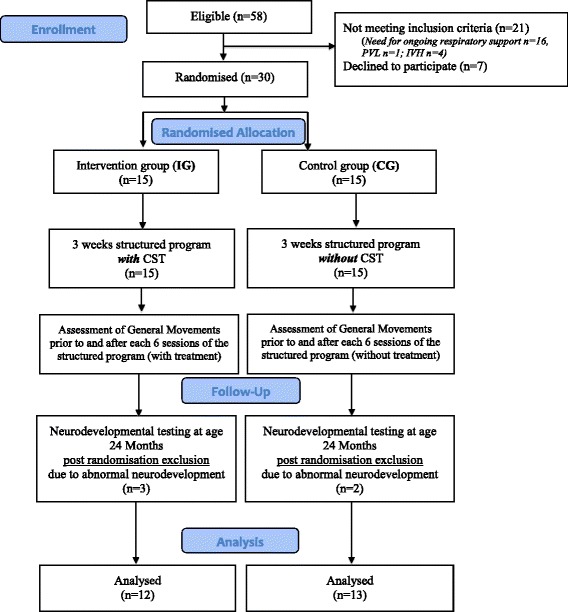
Flowchart and study design. CST = craniosacral therapy

Newborns were assigned to the intervention group (IG) or to the control group (CG) in a 1:1 ratio using a randomised block design with block size of 6. A sequentially numbered, sealed, opaque envelope containing the allocation was opened by a researcher after parental consent. Infants randomized to the IG received a total of six interventions. Interventions were standardised to 20 min/treatment with a frequency of two intervention/ week over three weeks. The CG did not receive the standardised intervention or any other kind of OMT interventions during this period (Fig. 1).

### Intervention

The intervention was performed by two specialized physiotherapists trained at the Upledger Institute Graz, Austria, with 10 years of experience and certified as Upledger CranioSacral® Therapists. The two physiotherapists involved in the study were trained to use only indirect and fluidic techniques. None of the physiotherapists were involved in the study design, data entry or statistical analysis. Both groups received usual care, which means – traditional (~routine) nursery care e.g. side lying and “nesting” in an incubator and skin to skin contact by the parents. In addition all parents, the physicians and the GM assessors were unaware of patient allocation except for the treating physiotherapist.

### Procedure

The intervention was scheduled according to the circadian rhythm of the infant (e.g. intervention was only started when the infant was awake and after feeding to avoid influence of agitation due to hunger). Infants were positioned in supine position, naked, wearing only swaddling bands. After initial contact with the child, by using the preferred initial touch, based on the requirements of basal stimulation, the specialized physiotherapist started with the evaluation of the craniosacral system according to a (modified) 10 step-program [7, 24]. The 10 step-program was modified as follows: exploration of the cranial system (step 1), treatment of asymmetry (step 2), evaluation of the overlapping of the cranial bones (step 4), exploration of the balance of the membranes of the cranial and spinal dura mater (step 7), exploration and treatment of the sacrum (step 8), and exploration and treatment of the chest (step 9). After the evaluation craniosacral therapy was initiated to achieve the greatest relaxation.

### Data collection

#### General Movements (GMs)

GMs are part of the spontaneous movement repertoire and are present from early fetal life onwards until the end of the first half a year of life. GMs are complex, occur frequently, and last long enough to be observed properly. They involve the whole body in a variable sequence of arm, leg, neck, and trunk movements. They wax and wane in intensity, force and speed, and have a gradual beginning and end. Rotations along the axis of the limbs and slight changes in the direction of movements make them fluent and elegant and create the impression of complexity and variability. If the nervous system is impaired, GMs lose their complex and variable character and become monotonous and poor [25]. The GMA has been validated with a specificity of 82-99 %, a sensitivity of 95-100 %, a negative likelihood ratio of 0.05, and a positive likelihood ratio > 51 to predict neuromotor development [25]

### Recording and analysis of GMs

The preterm infants were videotaped twice a week: in the IG 5 min before and after each intervention (which means before and after OMT), and same in the CG but without intervention.

The videos (10 min per session) were taken with the infants lying supine in the incubator wearing only diapers. Each infant was recorded 12 times, which corresponds to a 2-h footage per infant.

As the GMA is dependent on the behavioural state [26] of the infant, only infants in behavioural states 2 (i.e., eyes closed, irregular respiration, general movements present) or 4 (i.e., eyes open, irregular respiration, general movements present) were included. If an infant was in behavioural states 1, 3, or 5 (corresponding to quiet sleep, quiet wakefulness or crying), his/her GMs could not be assessed. Therefore, the number of infants varies slightly within the weekly assessments. All videos were edited according to the standards of GMA [27]. Normally, the GMs of a preterm infant comprise the entire body and manifest themselves in a variable sequence of arm, leg, neck and trunk movements. They appear and cease gradually, varying in intensity and speed. Rotations and frequent slight variations of the direction of motion make them look complex but smooth [15–18]. GMs are categorized as normal (N) or abnormal. Abnormal GMs are classified into (1) “poor repertoire GMs” (PR), whereby the sequence of movement components is monotonous; the amplitude, speed, and intensity lack the normal variability; (2) “cramped-synchronized GMs” (CS), which appear rigid as they lack the usual smoothness and fluent character; the limb and trunk muscles contract almost simultaneously and relax almost simultaneously; and (3) “chaotic GMs” (Ch), which appear jerky and abrupt due to their large amplitude and high speed [15, 18].

Besides the global GMA into the categories N, CS, PR, Ch (primary outcome) mentioned above, we assessed as detailed GMA, the General Movement Optimality Score (GMOS) as secondary outcome based on Prechtl’s optimality concept [17, 28]. The detailed GMA (GMOS) is a further tool to assess preterm motor movements. Thus it is a supplemental tool to define motor optimality with the principles of GMA. The GMOS is composed of the subscore for the sequence of movements (maximum = 2) and three sub-component optimality scores: (1) optimality for neck and trunk movements (maximum = 4); (2) optimality for the upper limb movements (maximum = 18); and (3) optimality for lower limb movements (maximum = 18). A higher score indicates a more optimal performance. For the GMOS, the maximum composite score of 42 indicates the most optimal GM performance. All GMs judgements were done by two experts in GMA (P.B.M., C.E.), who were blinded to group assignment, after completion of the 3-week structured program. In case of disagreement on particular details the recordings in question were re-evaluated until consensus on the final score was achieved. GMA has been validated as a diagnostic tool for detecting early brain dysfunction in newborn infants [19, 29–31]. The early identification of individual infants at high risk remains difficult. Spittle et al reviewed the clinometric properties of neuromotor assessments for preterm during the first year of life [16]. Furthermore, Bosanquet et al , Burger et al and Noble et al showed that Prechtl’s assessment of the quality of GMs offers the best combination of reliability, sensitivity and specificity for predicting cerebral palsy in the early months [19, 32, 33].

### Statistical analysis

To compare global GMA (N vs. PR vs. CS) between groups before the first session and after the last session Fisher’s exact test was used.

To analyze GMOS a linear mixed model with fixed effects for session, time point (time point refers to the comparisons of the measurements before vs. after each session) and intervention (IG vs. CG), random effect for individual children and a first order autoregressive covariance structure was used for calculation of significant differences between groups and interactions. Following interaction terms were included in the model: session*time point, session*intervention, time point*intervention and session*time point*intervention. Exploratory post hoc analyses (linear mixed model, fixed effects: session, time point and intervention, random effect: individual children, interaction: session*time point*intervention) were performed to determine group differences for all twelve measurement (before and after all six sessions) separately. To compare patient’s characteristics Pearson Chi Square test and Mann Whitney u-Test was used. To analyse inter-scorer agreement Cohen’s Kappa was calculated. A *p*-value of a *p* < 0.05 was considered statistically significant. Statistical analysis was performed using IBM SPSS Statistics 22 (SPSS Inc. Chicago, IL, USA, 2013).

## Results

Fifty-eight (58) newborn infants with a GA between 25-33 weeks were eligible. 28 could not be included because they did not meet the inclusion criteria (need for ongoing respiratory support *n* = 16, diagnosis of Periventricular Leukomalacia n = 1 and Intraventricular Haemorrhage *n* = 4) or the parents declined to participate (*n* = 7). 30 infants (male *n* = 16/female *n* = 14) were randomised to either IG (*n* = 15) or CG (*n* 15) and included in the study. All randomised infants underwent the three-week study protocol. All infants tolerated the intervention well without any side effects. Overall, five infants (IG *n* = 3, CG = 2) had to be excluded during neonatal follow-up. Four infants (three in the IG and one in the CG) developed cerebral palsy and one infant in the CG developed asymmetric muscular dystonia. Therefore, a total of 12 infants in the IG and 13 infants in the CG were available for final analysis (Fig. 1). Participants’ characteristics are presented in Table 1.

**Table 1:** Demographic data of all 25 included participants

	Intervention Group (IG)	Control Group (CG)	Pearson Chi Square
N (Male /Female)	12 (6/6)	13 (5/8)	*P* > 0.05
GA* at birth			
Median	28	30	*P* > 0.05
Range	25-33	27-33	
Birth weight(g)			
Median	1129	1170	*P* > 0.05
Range	690-1700	855-1760	
GA* at first video recording			
Median	31.5	33	*P* > 0.05
Range	31-35	30-34	

*GA gestational age in completed weeks

### General Movement Assessment (GMA)

Of 300 video recordings, 28 (9 %) had to be excluded, because the behavioural state of the infants did not allow proper assessment, and a further 4 (1 %) were excluded due to technical problems during recording. The footage for detailed analysis consisted of 268 assessable recordings and a total of 66 h recording time. The global GMA was done off-line from video and usually took the experienced observer 3-5 min per recording (Mean: 4 min; total 17.5 h of analysis). The detailed assessment (GMOS) was done offline from some video and lasted between 20 and 30 min per recording (Mean: 23 min; total 112 h of analysis).

Primary outcome: GMA

The GMA at the beginning of the interventional period showed that 5 infants had normal GMs (N) and 20 a poor repertoire of GMs (PR). There was no difference in global GMA between the two groups (IG: 1 N, 11 PR; CG: 4 N, 9 PR; Fisher’s exact Test *P* > 0.05). At the end of the interventional period GMA were again comparable between groups (IG: 2 N, 9 PR, 1 CS; CG: 5 N, 7 PR, 1 CS; Fisher’s exact Test *P* > 0.05). In each group one infant with a PR of GMs improved to N and one infant deteriorated from PR to CS.

Secondary outcome: GMOS

**Fig. 2:**
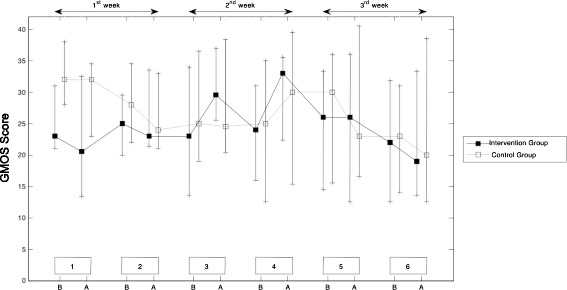
Secondary outcome: Course of GMOS in Intervention Group (IG) and Control Group (CG). GMOS did not change from session to session (main effect session: *p* =0.262) in either group. x-axis: 6 episodes of video assessment during study period of 3 weeks (B = Before Intervention, A = After Intervention). y-axis: GMOS Score. The symbols represent medians and the variations represent the interquartile (P25, P75)

GMOS did not change from session to session (main effect session: *p* = 0.262). Furthermore no differences between IG and CG (main effect group: *p* = 0.361) and no interaction of session*group could be observed (interaction session*group: *p* = 0.658) (Table 2). Post hoc analysis showed a trend toward higher values before (post hoc analysis for group differences at session 1, and time point 1: *p* = 0.085) and after (post hoc analysis for group differences at session 1, and time point 2: *p* = 0.075) the first session in CG compared to IG group. At all other time points GMOS were not significantly different between groups. (Table 3, Fig. 2).

**Table 2:** Effect of group (Intervention group, Control Group), session (six different sessions), time point (before and after each session) and interaction terms on General Movement Optimality Score. (Linear mixed model, Type III Tests of Fixed Effects)

Source	Numerator df	Denominator df	F	*P*
Intercept	1	30.899	336.764	0.000
Group	1	30.899	0.859	0.361
Session	5	192.761	1.308	0.262
Time point	1	235.000	1.691	0.195
Group * Session	5	192.761	0.655	0.658
Group * Time point	1	235.000	2.151	0.144
Session * Time point	5	205.358	2.042	0.074
Group * Session * Time point	5	205.358	0.501	0.775

**Table 3:** Study outcomes

	Intervention Group (IG)	Control Group (CG)	sign.
PRIMARY OUTCOME			
GMA	1 N, 11 PR;	4 N, 9 PR;	Fisher’s exact Test
before the 1^st^ session			*P* > 0.05
Global GMA	2 N, 9 PR, 1 CS;	5 N, 7 PR, 1 CS;	Fisher’s exact Test
after the last session Assessment			*P* > 0.05
SECONDARY OUTCOME			
GMOS	23 (21-31)	32 (28-38)	P* = 0.085
before the 1^st^ session			
Median (Interquartile Range)			
GMOS	19 (13.5-33.3)	20 (12,5-38.5)	P* = 0.722
after the last session			
Median (Interquartile Range)			

P* values from exploratory post hoc analyses followed linear mixed model with a fixed effect for session, time point (before vs. after session) and intervention (IG vs. CG)

*N* normal General Movements

*PR* poor repertoire General Movements

*CS* cramped-synchronized General Movements

### Inter-observer agreement

For the GMA (primary outcome) the inter-scorer agreement revealed a Cohen’s Kappa of 0.87; for the GMOS (secondary outcome) Cohen’s Kappa was 0.76.

## Discussion

To the best of our knowledge this is the first randomized controlled trial using the non-intrusive GMA to evaluate short-term effects of craniosacral therapy in preterm infants who have been admitted to the neonatal intensive care unit.

No difference in the GMA (primary outcome) during the whole intervention period could be observed between groups.

Overall the GMOS (-secondary outcome) i) did not change over time; ii) showed no differences between IG and CG; iii) showed comparable courses in the two groups; and iv) a post hoc analysis showed a trend toward higher values before and after the first session in CG, whereas at all other time points GMOS were not significantly different between groups (Table 2, Fig. 2).

Craniosacral therapy is one of the most careful and non-invasive therapies of OMT and one of the most popular non-pharmacologic complementary therapies in adult medicine [34, 35]. In addition, there are some data available from infants and children [36, 37], reporting positive effects of craniosacral therapy [38] and cranial osteopathy in gastrointestinal function [39], obstructive apnea [40] and postural asymmetry [41]. In addition, two clinical trials have been carried out in preterm infants while admitted to the neonatal intensive care units. [8, 42]. Pizzolorusso et al evaluated the effect of indirect and fluid OMT techniques on 352 infants suggesting that this intervention potentially reduces LOS (adjusted OR = 0.45; 0.26-0.74) [8]. In addition, this observational study reported a significant reduction in number of episodes of vomiting, regurgitation, gastric residual and enema (adjusted OR = 0.22; 0.09-0.51) [8]. Cerritelli et al investigated the effect of OMT in preterm infants and reported a significant reduction in LOS. Infants who received OMT had a mean LOS of 26.1 ± 16.4 days compared to 31.3 ± 20.2 days in the control group (*p* < 0.03) [37, 43].

However, the effects of OMT and craniosacral therapy in preterm infants are not fully understood [8, 10, 37, 38]. There are several hypotheses including i) anti-inflammatory effects, ii) increases in the opioid reactions, or iii) effect on the autonomic nervous system. Narendran et al reported higher level of cortisol, albumin, IL-8 and IL-1β in preterm infants [37, 44], suggesting an increased level of systemic inflammation. A recent study demonstrated that osteopathic treatment could reduce the inflammatory process acting mainly on anti-inflammatory factors [37, 45]. In addition, Degenhardt et al suggested that OMT could have a role in increasing the opioid reaction [37, 46]. However, this hypothesis has some intrinsic limitations in terms of the sample used, i.e. no infants were osteopathically treated, and in translating in vitro findings into in vivo mechanisms. Longin et al [37, 47] reported that the gestational age of newborn infants is correlated with changes in heart rate variability (HRV). From the osteopathic perspective, changes in the HRV were recorded after the application of myofascial release techniques [37, 48, 49]. For this reason, the application of OMT could balance the sympathetic and parasympathetic inputs, creating an improvement of newborns clinical condition [8, 10].

GMs can be first seen in foetuses as young as 9 weeks post-menstrual age. Initially they are called foetal GMs and can be investigated during ultrasound recordings. Up to term age birth they become preterm GMs [15]. GMA is a new technique, which is based on the investigation of spontaneous movements, introduced by Prechtl et al [16–20]. Using the global GMA (primary outcome), PR GMs were observed most frequently in the present study, which is similar to previously reported findings [50]. A high incidence of PR GM’s is known in preterm infants and does not necessarily result in neurodevelopmental deficits. Because of that, the predictive value of the quality of GMs soon after birth is largely unknown and the predictive power of PR GMs is low [15, 46, 51]. On the other hand, PR GMs clearly demonstrate that the infant’s nervous system is not in an optimal condition at the time of recording.

Two of the infants (1 IG and 1CG) showed CS GMs during the study period, but developed normally over time. CS GMs, the most severe motor abnormality, has been found to be predictive of severe neurological impairment but only if they are consistent over time or predominant from preterm birth to 5 months post term age. If CS GMs appear transient their predictive value is low and normal development will follow in most cases [52]. The GMOS (detailed GMA = secondary outcome) did not change over time (*p* = 0.262) in both the IG and the CG,

### Strengths of the study

We would like to emphasize that i) only infants with normal and adequate neurodevelopmental evaluation remained within the study groups; therefore, all included infants (in both groups) showed adequate normal neurodevelopment at the age of two years, ii) this was a randomised trial, iii) we used an established assessment instrument (general (primary outcome) and detailed GMA (secondary outcome) and iv) the assessors of the GMs were completely blinded with respect to group assignment. In addition, there was no difference in terms of their diseases of prematurity, the diagnosis of respiratory distress or bronchopulmonary dysplasia [53] or the duration of mechanical ventilation.

### Weaknesses of the study

We only included a small sample size, which is a limitation of the study. We planned enrollment of 12 subjects per group, as proposed by Julious [54] and Billingham et al [55]. An additional 20 % (total of 15) was included for each treatment arm, anticipating subject withdrawal or other unforeseen postenrollment exclusions from the study [54, 55]. Based on our results we conducted power analysis and sample size calculation. Our study has a power of 22 % to detect an interaction of intervention*time. In a future trial using the same approach, 57 children in each group would have to be included to detect a significant treatment effect (interaction of intervention*time) with a power of 80 %.

The following questions arise: Are two interventions per week enough to see any significant effects? Would the results be different by using another kind of OMT? Should further studies include a control group with “touch” and “presence” of the therapist?

Nevertheless, the results of the present study underline the fact, that using craniosacral therapy in preterm infants is safe, as there was no deterioration of neurodevelopmental results in IG.

## Conclusion

In conclusion, we were able to show that a group of “healthy” preterm infants undergoing an intervention with craniosacral therapy (IG) showed no significant changes in global (primary outcome) and detailed GMA (secondary outcome) during an observation period of 3 weeks compared to infants without intervention (CG). In view of the fact that the GMOS (secondary outcome) did not deteriorate in the IG, craniosacral therapy seems to be safe in preterm infants.

Currently the treatment of preterm infants with any kind of OMT should be limited to clinical trials. Studies evaluating short- and long-term effects are urgently needed. Furthermore, future studies should include a control for touch and presence of the therapist.

## Abbreviations

CAM: Complementary and alternative medicine
OMT: Osteopathic manipulative treatment
LOS: Length of Hospital Stay
GA: Gestational Age
TIMP: Test of Infant Motor Performance
GMs: General movements
N: normal General Movements
PR: poor repertoire General Movements
CS: cramped-synchronized General Movements
Ch: chaotic General Movements
GMA: General Movement Assessment (global GMA)
GMOS: General movement optimality score (detailed GMA)
